# Editorial: Prosocial and Antisocial Behavior in Economic Games

**DOI:** 10.3389/fnbeh.2016.00243

**Published:** 2016-12-26

**Authors:** Pablo Brañas-Garza, Antonio M. Espín, Benedikt Herrmann, Praveen Kujal, Rosemarie Nagel

**Affiliations:** ^1^Economics Department, Business School, Middlesex University LondonLondon, UK; ^2^Faculty of Social Sciences, School of Economics, University of NottinghamNottingham, UK; ^3^Department of Economics, ICREA, Universitat Pompeu Fabra, Barcelona Graduate School of EonomicsBarcelona, Spain

**Keywords:** prosocial behavior, antisocial behavior, economic games, cognition, fMRI, neuroscience of decision making

In this Research Topic, a collection of research and review articles contribute to our understanding of the factors influencing human prosocial and antisocial behavior in economic games. Under the labels of “prosocial” and “antisocial” behavior we consider all those actions that help or hurt others, respectively. While the prosocial, bright side of human behavior has received much attention in more than two decades, its antisocial, dark side is far less studied. This Research Topic aims to combine both sides into a comprehensive account of human social behavior.

The games that are used in the different studies are typically derivations of well-known and much studied setups such as the Public Goods, Ultimatum, Trust and Dictator Games, all invented in the 80's and 90's. However, many of our discussed articles include new measures and techniques from biology and neuroscience (e.g., digit ratio, fMRI, skin conductance) or psychology (e.g., cognitive reflection, self-control, meditation) which in the first decades of the study of social behavior using economic games were alien to economists. This could only happen through a strong and fruitful interaction between economists, psychologists, biologists and neuroscientists in the past 15 years.

Several articles in the Research Topic have focused primarily on prosocial behaviors discussing concepts like altruism, cooperation, fairness and efficiency (typically in opposition to pure self-interest). These include Rand and Kraft-Todd, Schniter and Sheremeta, Jaber-López et al., Volz et al., Raihani and Bshary, Galizzi and Nieboer, Kuss et al., Espinosa and Kovarik, Gesiarz and Crockett, Arruñada et al., Bejarano et al., Clots-Figueras et al., and Chierchia and Coricelli.

Others have also added analyses or discussions about antisocial behaviors, introducing notions such as punishment, envy, spite or intergroup competition. These include Chen and Perc, McCall et al., Nash et al., Zhu et al., Everett et al., Azar et al., Cárdenas and Mantilla, Hu et al., Fatás and Mateu, Ponti and Rodríguez-Lara, Espín et al., Corgnet et al., Andrighetto et al., and Fischer et al.

One particularly prominent strand of this research has studied the cognitive basis of prosocial and antisocial behaviors building on the distinction between automatic/intuitive and controlled/deliberative cognitive processes (i.e., from a dual-process perspective). Rand and Kraft-Todd find that individuals cooperate more in a one-shot Public Goods Game (PGG) when they are forced to decide quickly, which prompts intuitive decision-making, compared to a “deliberative” condition where they are forced to delay their choices. In addition to this result, which corroborates previous findings (e.g., Rand et al., 2012, 2014), they find that when the payoff structure of the PGG is manipulated in such a way that cooperation is payoff-maximizing, the effect of intuition vs. deliberation no longer exists. These findings are in line with the Social Heuristics Hypothesis developed in Rand et al. (2014), which states that humans internalize social behaviors that are beneficial in real-life long-run interactions and apply them intuitively to one-shot encounters where they are disadvantageous. Kuss et al. classify subjects according to their social value orientation (Van Lange et al., 1997) into proselfs and prosocials and analyze the brain activation patterns during money-allocation decisions using functional magnetic resonance imaging (fMRI). When being prosocial is not costly, the authors find that prosocial choices are associated with increased activation in the ventromedial and dorsomedial prefrontal cortices, especially in the proself sample. These results are consistent with the argument that prosocial decisions in those classified as prosocials are more intuitive, whereas they demand more active deliberation in proself individuals. Relatedly, Espinosa and Kovarik reanalyze data from previous experiments and suggest that gender may also moderate the connection between cognitive processing and prosocial behavior: encouraging deliberation/reflection seems to decrease the prosociality of males but not females.

To the extent that strategic reasoning may be related to reflection (e.g., Brañas-Garza et al., 2012), the results of Arruñada et al. also provide interesting insights into the cognitive basis of social behavior. The authors find that strategic reasoning capacity is unrelated to prosocial vs. selfish choices at the individual level.

From a neuroscientific perspective, Nash et al. review previous evidence on the role of self-control for both prosocial and antisocial (i.e., punishment) decisions in an attempt to identify stable individual neural differences (mainly using resting-state electroencephalography and structural magnetic resonance imaging) that may account for individual differences in social preferences at the trait level. This review highlights the importance of the lateral prefrontal cortex in encoding cooperation and punishment behaviors. Along these lines, Corgnet et al. and Ponti and Rodríguez-Lara perform a trait-level analysis of the relationship between (distributional) social preferences and cognitive reflection, as measured by the Cognitive Reflection Test (Frederick, 2005; Toplak et al., 2014). Although following different methodologies, both studies arrive at similar conclusions: a more deliberative (vs. intuitive) cognitive style is related to a lower influence of “envy” (i.e., aversion to disadvantageous inequality) in decision-making. The relationship between cognitive styles and “compassion” (i.e., aversion to advantageous inequality), however, is more complex. These findings are consistent with a link of deliberation with “mild altruism” and self-interest, and of intuition with both egalitarian and spiteful preferences. Moreover, these relationships may help explain why a number of previous studies analyzing participants' single decisions in economic games have reported seemingly inconsistent findings (Corgnet et al.).

Gesiarz and Crockett provide a novel framework inspired by research on reinforcement learning in which social behavior stems from the interaction between three decision-making systems: a goal-directed system that selects actions based on their predicted consequences, a habitual system that selects actions based on their reinforcement history, and a Pavlovian system that emits reflexive responses based on evolutionarily prescribed priors. This three-system framework, although it shares many similarities with classical dual-process models in psychology, offers a promising new perspective to rationalize previous findings for which dual-process theory may fall short in providing an explanation.

Another prolific area of research in the Research Topic has focused on sanctioning (i.e., rewarding cooperators or punishing non-cooperators) behaviors and institutions. Previous research has highlighted the pivotal role of sanctions for the establishment of cooperation among humans (e.g., Yamagishi, 1986; Fehr and Gächter, 2002; Cuesta et al., 2008; Rand et al., 2009). Using evolutionary game theory, Chen and Perc derive the conditions for institutional reward and punishment to maximize cooperation in the PGG under scale-free networks. Their results indicate that both the strength of the synergetic effects of group interactions and the role of the sanctioned individuals' in the social network (i.e., how influential they are, as measured by the degree of the node they occupy) need to be considered for the design of optimal sanctioning institutions.

In a series of finitely-repeated PGG experiments, Fischer et al. compare the capacity of centralized and decentralized punishment institutional regimes for sustaining cooperation under different information conditions. Both punishment regimes lead to similar aggregate behavior when information about others' cooperation is either perfect or extremely noisy. However, at intermediate levels of noise, decentralized punishment erodes efficiency—but not cooperation—compared to the centralized regime partly due to the higher prevalence of spiteful, “antisocial punishment” (Herrmann et al., 2008) targeted at cooperators. In general, antisocial punishment is rare when one randomly selected group member monopolizes the power to punish, whereas it is often observed when all group members can punish each other. Fatás and Mateu show that the payoff structure of the cooperation mechanism may also modulate the presence of (decentralized) antisocial punishment and study how this affects cooperation in a finitely-repeated experiment. They compare the standard PGG framework, where the group profit is determined by the linear aggregation of cooperation levels (the average contribution to the group account), with the Weakest-Link Mechanism (WLM), where the group profit is a multiple of the minimum contribution in the group. The results demonstrate that the presence of antisocial punishment prevents the establishment of cooperation and reduces efficiency in the standard PGG but not in the WLM, in which antisocial punishment is rare. Another type of punishment that can hamper rather than enforce cooperation is counter-punishment (Nikiforakis, 2008). Andrighetto et al. conducted finitely-repeated PGG experiments with and without communication between participants where punishment and counter-punishment were possible. Their results show that given fixed identifiers, so that group members can track each other's behavior across periods, the key factor is the presence of communication. Communication leads to increases in cooperation and efficiency, regardless of counter-punishment opportunities.

Regarding the psychology of sanctioning behavior, Espín et al. study whether punishment in a large-scale one-shot Ultimatum Game (UG) experiment can be predicted by the participants' preferences for short-run vs. long-run outcomes (i.e., their intertemporal preferences). Previous results indicate that more impatient, short-run oriented individuals are more willing to reject low offers (i.e., punish the proposer) as responders in the one-shot UG (Crockett et al., 2010). Since the punishment of low offers in the UG can arise from both fairness and spiteful motivations (e.g., Brañas-Garza et al., 2014), Espín et al. analyze whether “impatient punishers” are themselves fair or unfair when playing as proposers in the game. Higher impatience predicts a higher likelihood of both punishing low offers as responders and making low offers as proposers, which suggest that the short-run motivation in the UG is to spitefully reduce the other player's payoff rather than equalize the players' payoffs (i.e., fairness). This result mimics previous evidence from the one-shot PGG where more impatient individuals were more likely to (spitefully) punish non-cooperative group members while being non-cooperative themselves (Espín et al., 2012).

McCall et al. analyze experimentally the effect of long-term compassion meditation on punishment behavior in a one-shot game similar to the UG. They find that, compared to controls, compassion meditators are less likely to punish stingy proposers, suggesting that punishment has a strong spiteful component in second-party situations such as the UG, in line with the arguments of Espín et al. However, compassion meditators and controls do not differ in their willingness to punish low offers received by others when acting as third-party punishers, which indicates that third-party punishment is more influenced by moralistic norm-enforcement and less influenced by spite or vengeance, compared to second-party punishment (Crockett et al., 2013). Finally, McCall et al. also observe that compassion meditators are more likely than controls to compensate the victim when given the opportunity as third-party observers.

Using fMRI, Hu et al. investigate the neurobiological underpinnings of third-party punishment and compensation behavior in one-shot interactions. They find that both behaviors are associated with activation in the bilateral striatum, which suggests that people may derive satisfaction from both punishing the norm-violator and compensating the victim. In addition, the observed functional connectivity between the bilateral striatum and the lateral prefrontal cortex during both types of decisions suggests that paying a cost to punish the violator or to compensate the victim requires controlling a selfish impulse. The ventral medial prefrontal cortex, however, is differentially connected with the bilateral striatum during punishment, suggesting that the integration of emotional information might play a more important role in punishment than in compensation decisions.

Intergroup competition has attracted a non-negligible share of attention in this Research Topic. The desire to outcompete other groups is considered one of the possible motivational factors driving cooperation in humans, a pattern of behavior that seems to have solid evolutionary roots (Henrich, 2004). Cárdenas and Mantilla run finitely-repeated PGG experiments with and without intergroup competition. In both treatments subjects receive feedback about their relative performance within their group and about the relative performance of their group with respect to other groups. In the treatment with intergroup competition, subjects belonging to high relative performance groups earn extra money whereas without intergroup competition the feedback on groups ranking is merely informative. Even though intergroup competition increase within-group cooperation in line with the equilibrium predictions, the authors find that individuals with low relative performance reduce their contributions to the public good while groups with low performance increase theirs, regardless of the treatment.

Zhu et al. observe that children as young as 2.5–3.5 years already display ingroup favoritism, and to a larger extent than 5.5–6.5 year-olds. However, the older group is more fairness-oriented (i.e., more egalitarian) than the younger group when intergroup competition is absent, which suggests that as children age ingroup favoritism is substituted by a more generalized sense of fairness.

In a thorough literature review on ingroup favoritism in economic games, Everett et al. distinguish between the preferences approach and the beliefs approach and conclude that both types of considerations seem to matter. Thus, individuals may favor their group because their social preferences change according to group membership (ingroup love, outgroup derogation) but also because they expect more reciprocal behavior from ingroup than outgroup members. Moreover, both types of considerations may reinforce each other (e.g., stereotypes, as one form of beliefs, may shape preferences) and whether one or the other consideration dominates could depend on a number of factors.

Azar et al. explore beliefs in a multi-period UG experiment where participants face a different partner in each period. The authors find that even though proposers' beliefs about the minimal acceptable offer does not change following a rejection, they increase their offers if they were rejected in the previous period. Furthermore, responders are more likely to reject an offer when the offer falls short of either their expectations about the amount that will be offered to them or the minimal amount they believe other responders will accept. Beliefs are also key for understanding behavior in situations such as the Trust Game (TG) and Coordination Games. Schniter and Sheremeta study how communication and emotions shape trust and trust re-extension in a (unexpected) second TG interaction. They find that emotions triggered by interaction outcomes are predictable and also predict subsequent apology and trust re-extension. The role of emotions in behavioral regulation may help explain why communication is produced, when messages can be trusted, and when trust will be re-extended. In one-shot Coordination Game experiments, Chierchia and Coricelli observe that expected payoffs, and under certain conditions also the likelihood of coordination, tend to increase with the level of (experimentally induced) perceived similarity between the players. Further experiments suggest that the observed effects should not be reduced to liking similar others (“homophily”) but it is also about predicting them.

In one-shot TG with uncertainty about the benefits from trust (i.e., the trustor does not know by how much the trusted amount is increased before reaching the trustee), Clots-Figueras et al. study how trustees use deception to influence the trustor's behavior. They find that about two-thirds of trustees send false messages about the benefits from trust and half of them are believed by the trustor. However, some trustees lie to benefit themselves whereas others lie to increase the pie and then share it equally with the trustor.

Volz et al. explore the neural basis of deception in the TG using fMRI and observe that the right temporo-parietal junction, the dorsal anterior cingulate cortex, the (pre)cuneus, and the anterior frontal gyrus encode the contrast between lying and truth telling. Further contrasts show that brain activation can reveal an individual's veridical intention to deceive others in order to increase her own payoff, regardless of whether deception involves lying or telling the truth when the message is expected not to be believed (i.e., sophisticated deception). Jaber-López et al. experimentally analyze the behavioral and physiological patterns of corruption. They find that many people are prosocial and avoid corrupt, unethical behaviors even when they could benefit from them. Physiologically, the participants' skin conductance responses findings suggest that stronger emotions are associated with decisions deviating from pure money maximization, rather than with (un)ethical behavior *per se*.

Galizzi and Nieboer replicate previous findings (Brañas-Garza et al., 2013) on the relationship between 2D:4D digit ratio (a biomarker of the relative prenatal exposure to testosterone vs. estrogens) and altruism in a Caucasian sample. In particular, individuals with an intermediate digit ratio give more money in a Dictator Game than individuals with either low or high digit ratios. However, this result does not hold in non-Caucasian samples, which may suggest an interaction between environment and hormones in shaping altruistic behavior.

Raihani and Bshary critically review previous theories on the evolution of human altruism. In particular, they confront the “mismatch” (Cosmides and Tooby, 1989) and the cultural group selection (Henrich, 2004) hypotheses. The “mismatch” hypothesis argues that behavior is constrained by psychological mechanisms which evolved predominantly in the context of repeated interactions with known and/or genetically-related individuals. In contrast, cultural group selection posits that humans have been selected to cooperate in anonymous one-shot interactions due to strong intergroup competition, which creates interdependence among ingroup members. Raihani and Bshary further discuss alternative routes by which humans could increase their direct fitness by cooperating with strangers under natural conditions.

Finally, Bejarano et al. observe prosocial behavior in an experiment on lifetime investments in enjoyment. Individuals were permitted to freely chat in two different treatments: an individual's rewards from investments in life enjoyment depend either only on her choices or also on their similarity to the choices of others, generating a premium on conformity. Incentives for conformity appear to promote prosocial behavior, but also increase variance among groups, leading to convergence on suboptimal strategies for some groups.

## Author contributions

All authors listed, have made substantial, direct and intellectual contribution to the work, and approved it for publication.

## Conflict of interest statement

The authors declare that the research was conducted in the absence of any commercial or financial relationships that could be construed as a potential conflict of interest.
